# miR-874-3p is down-regulated in hepatocellular carcinoma and negatively regulates PIN1 expression

**DOI:** 10.18632/oncotarget.14526

**Published:** 2017-01-05

**Authors:** Ka-Wai Leong, Chi-Wai Cheng, Chun-Ming Wong, Irene Ng Oi-Lin, Yok-Lam Kwong, Eric Tse

**Affiliations:** ^1^ Department of Medicine, The University of Hong Kong, Hong Kong; ^2^ Department of Pathology and State Key Laboratory for Liver Research, The University of Hong Kong, Hong Kong

**Keywords:** micro-RNA, miR-874-3p, peptidyl-prolyl-isomerase, PIN1, hepatocellular carcinoma

## Abstract

PIN1 is a peptidyl-prolyl *cis/trans* isomerase (PPIase) that regulates multiple signaling pathways to control cell fate and is found to be over-expressed in cancers, including hepatocellular carcinoma (HCC). However, the regulation of PIN1 in HCC remains poorly defined. Micro-RNAs (miRNAs) have been reported to play a pivotal role in oncogenesis by targeting the 3′-untranslated region (UTR) of mRNAs encoded by oncogenes and tumour suppressor genes, thereby suppressing the levels of both oncoproteins and tumour suppressors. In this report, we aimed to identify miRNAs that suppress PIN1 expression and to determine their role in HCC. By searching the TargetScan database, miR-874-3p was identified as a potential negative regulator of PIN1. miR-874-3p was demonstrated to bind the 3′UTR of *PIN1* mRNA directly to suppress expression of PIN1. Functionally, over-expression of miR-874-3p in HCC cell line PLC/PRF/5 inhibited cell growth and colony formation *in-vitro*, and promoted cellular apoptosis. Furthermore, these tumour suppressive functions conferred by miR-874-3p were abrogated by over-expression of PIN1. Similarly, expression of miR-874-3p in PLC/PRF/5 with PIN1 knocked-down did not further suppress cellular proliferation, suggesting that PIN1 was a major target of miR-874-3p. More importantly, miR-874-3p was found to be down-regulated in HCC tissues and its expression was negatively correlated with that of PIN1. Down-regulation of miR-874-3p was also associated with poorly differentiated tumour cells, more advanced staging, and inferior patient outcomes. In addition, over-expression of miR-874-3p suppressed tumour growth *in vivo*. Taken together, our data suggested that miR-874-3p plays a tumour suppressive role in HCC through down-regulation of PIN1.

## INTRODUCTION

Peptidyl-prolyl *cis*-*trans* isomerase NIMA-interacting 1 (PIN1) is an enzyme that, through its WW domain, binds to proteins with specific phosphorylated serine or threonine residues preceding proline (pSer/Thr-Pro), leading to their conformational and functional changes [1]. This is mediated by isomerization of the pSer/Thr-Pro peptide bonds with its prolyl isomerase (PPIase) domain, resulting in alteration of the activity, stability, protein-protein interaction and sub-cellular localization of these proteins [2, 3]. Because of these functions, PIN1 modulates many key cellular processes, such as cell cycle progression, cell proliferation and apoptosis. Consequently, dysregulation of PIN1 may result in tumour development [4–6]. Over-expression of PIN1 is found in many cancers, including hepatocellular carcinoma (HCC) [7]. We have demonstrated that PIN1 is over-expressed in over 50% of HCC and its over-expression leads to β-catenin and cyclin D1 accumulation in tumour cells [8]. Moreover, mouse xenograft experiments confirmed that PIN1 over-expression contributes to hepatocarcinogenesis and enhances the oncogenic function of the hepatitis B virus x-protein (HBx) in HCC [9, 10]. In addition, PIN1 also inhibits apoptosis in HCC by enhancing the anti-apoptotic function of survivin [11]. These important and diverse functions of PIN1 in promoting the malignant properties of HCC cells suggest that therapeutic intervention targeting PIN1 may be efficacious in HCC. Despite the biologic and potential therapeutic importance of PIN1 in HCC, the regulation of its expression remains poorly understood. The human *PIN1* gene is located in chromosome 19, and there is no evidence yet to suggest that the *PIN1* gene is amplified in cancers [12]. One of the early studies has shown that PIN1 level is promoted by the retinoblastoma/E2F pathway [13]. E2F proteins bind to the *PIN1* promoter and activate gene transcription. It has been postulated that the frequently dysregulated retinoblastoma pathway is the cause of PIN1 over-expression in breast cancer [13]. However, whether this proposition is valid in other cancer types remains to be defined.

MiRNAs are small non-coding RNAs that regulate gene expression at both transcriptional and post-transcriptional levels [14]. Aberrant expression of miRNAs has been shown to be associated with pathogenesis of cancers [14]. While some miRNAs are oncogenic in nature (oncomir), there are miRNAs that possess tumour suppressive action, and their levels are decreased in cancer cells. In HCC, a global reduction of miRNAs expression is tightly associated with tumour progression [15]. Many of these “tumour-suppressive” miRNAs suppress the expression of genes that positively promote tumour development and progression. More recently, miRNAs-miR-200c and miR-296-5p have been shown to inhibit PIN1 expression in breast and prostate cancer, respectively [6, 16]. However, no miRNA has been reported to reduce PIN1 expression in HCC.

To identify miRNAs that may regulate PIN1 expression in HCC, we performed an initial search (TargetScan 6.2) and identified 102 miRNAs targeting the 3′-untranslated region (UTR) of *PIN1* mRNA. Six potential miRNAs (miR-296-5p, miR-874-3p, miR-4665-3p, miR-3173-5p, miR-1587-5p and miR-1207-5p) with the highest total context score were selected for further testing in this study.

## RESULTS

### Down-regulation of PIN1 suppressed cell proliferation and colony formation, and induced apoptosis in HCC cell lines

**Figure 1:**
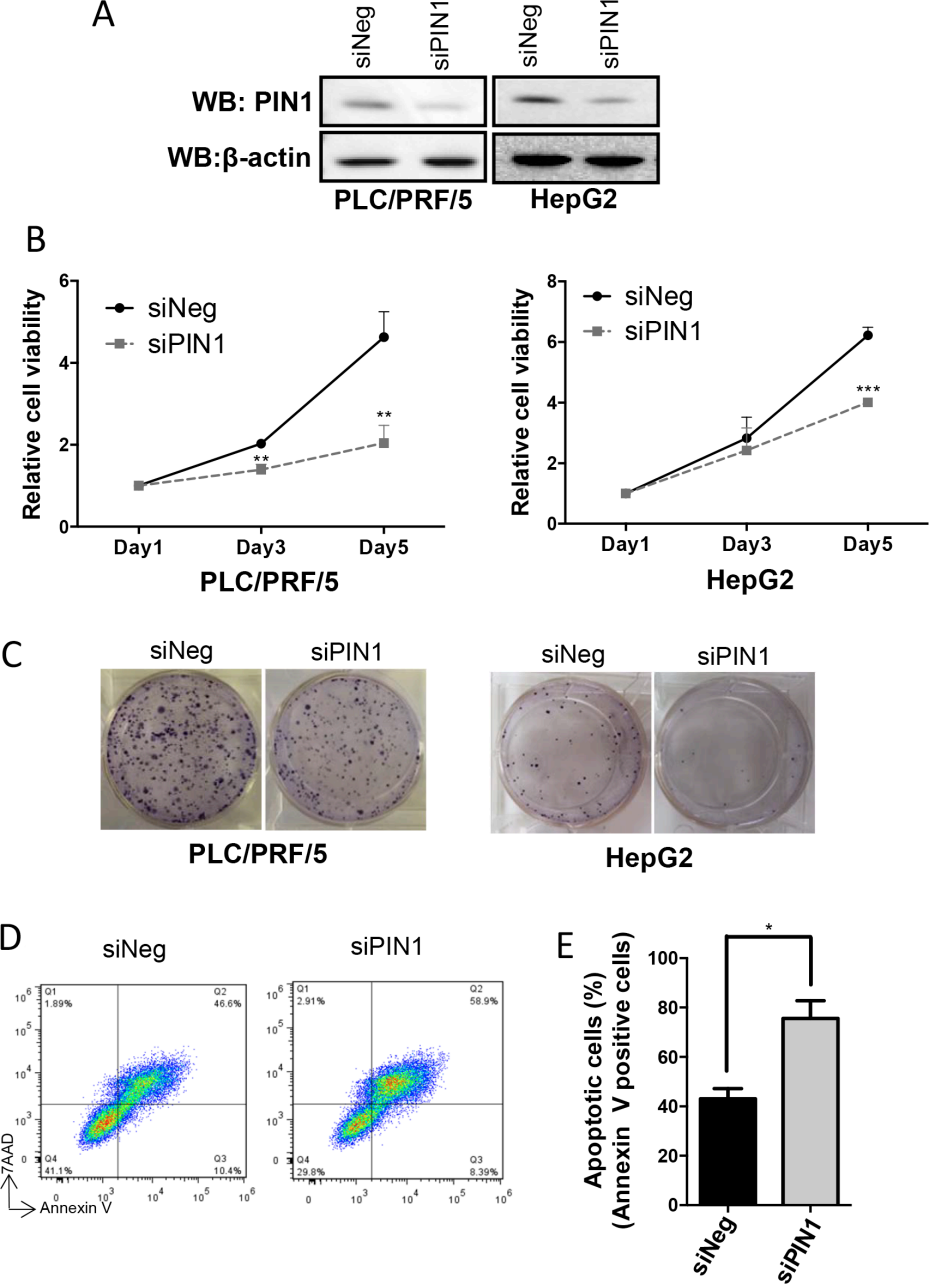
Down-regulation of PIN1 suppressed cell proliferation and colony formation, and enhanced apoptosis of HCC cell lines (**A**) Western immunoblots showing down-regulation of PIN1 by siRNA in HepG2 and PLC/PRF/5. β-actin was used as an internal control. (**B**) PIN1 knock-down by siRNA suppressed proliferation of HepG2 and PLC/PRF/5 cells as shown by MTT assay. (**P ≤ 0.01, and ***P ≤ 0.001, paired t-test) (**C**) In-vitro assay showing significant suppression of colony formation by HepG2 and PLC/PRF/5 cells after PIN1 knocked-down. (**D**) FACS analysis scatter plots showing the increase in apoptosis of PLC/PRF/5 cells (positive staining for annexin-V) after PIN1 knocked-down. (**E**) Diagram illustrating the significant increase in apoptotic cells after PIN1 knocked-down in PLC/PRF/5. Three independent sets of experiments were performed. (*P ≤ 0.05, paired t-test).

Specific small interfering RNA (siRNA) was used to suppress *PIN1* expression (Figure 1A). Down-regulation of PIN1 expression resulted in decreased proliferation and *in-vitro* colony formation of PLC/PRF/5 and HepG2 cells (Figure 1B and 1C). Moreover, PIN1 depletion also enhanced staurosporine(STS)-induced cellular apoptosis (Figure 1D and 1E). These results confirmed and validated that growth and survival of HCC cells were positively modulated by PIN1 expression.

### miR-296-5p and miR-874-3p decreased PIN1 expression

**Figure 2:**
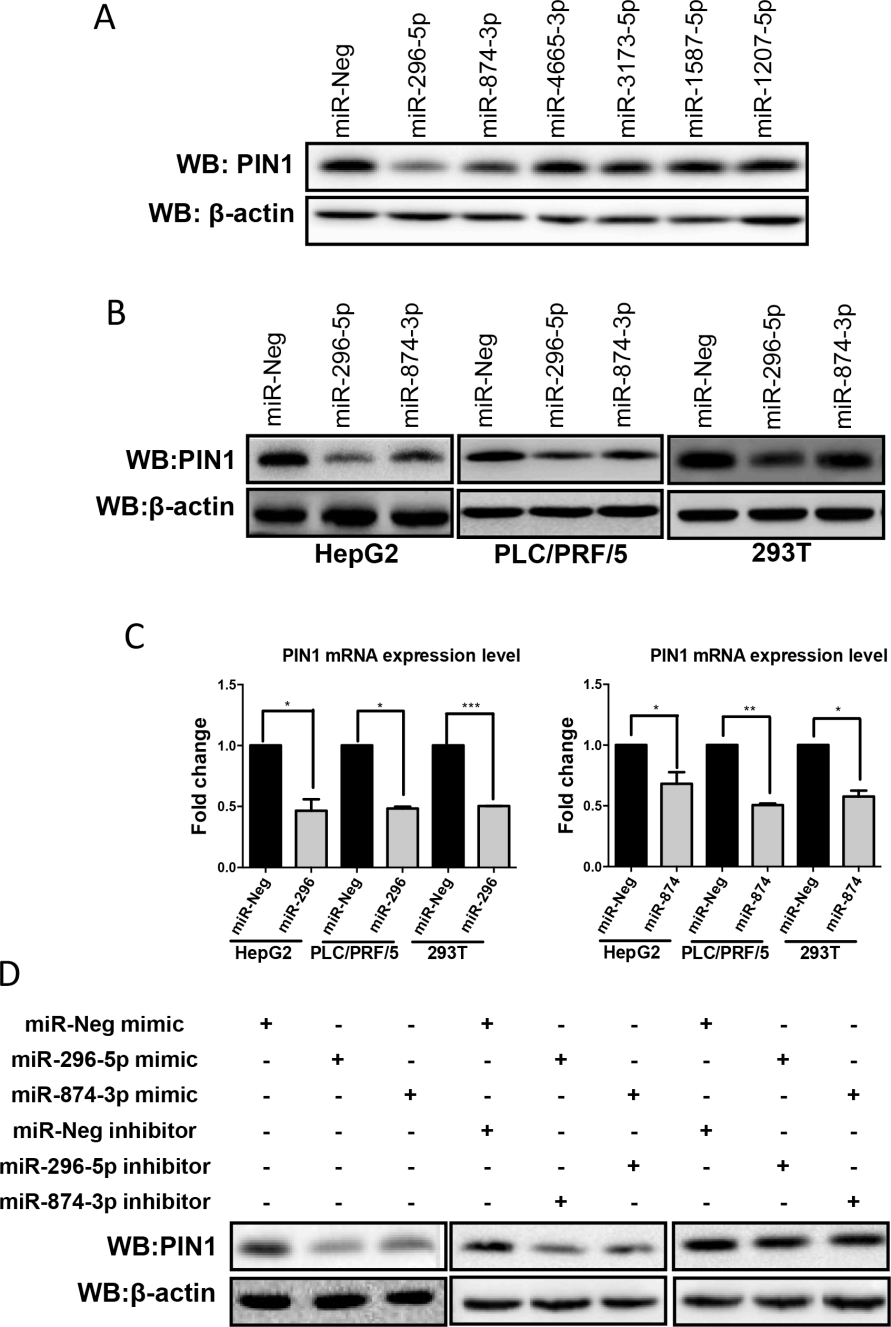
Over-expression of miR-296-5p and miR-874-3p decreased PIN1 mRNA and protein expression levels (**A**) Of the 6 miRNAs tested, only miR-296-5p and miR-874-3p suppressed PIN1 expression in HepG2 cells as examined with western immunoblot. (**B**) Western blot showing PIN1 down-regulation in all 3 cell lines transfected with miR-296-5p and miR-874-3p (**C**) PIN1 mRNA was suppressed by miR-296-5p (left panel) and miR-874-3p (right panel) as determined by RT-qPCR. (*P ≤ 0.05, **P ≤ 0.01, and ***P ≤ 0.001, paired t-test) (**D**) Western blots showing that miR-296-5p and miR-874-3p inhibitors abolished the PIN1 regulation induced by their corresponding miRNAs.

Initial search revealed that 102 miRNAs may target *PIN1* mRNA 3′UTR to regulate PIN1 expression (TargetScan 6.2). Six potential miRNAs (miR-296-5p, miR-874-3p, miR-4665-3p, miR-3173-5p, miR-1587-5p and miR-1207-5p) were selected for further experiments based on their high total context scores. Among them, only miR-296-5p and miR-874-3p were found to significantly down-regulate PIN1 protein in HCC cell lines HepG2 and PLC/PRF/5 (Figure 2A and Supplementary Figure 1). To further validate the effect of these miRNAs in PIN1 down-regulation, miR-296-5p and miR-874-3p were transiently transfected into HepG2, PLC/PRF/5, and human embryonic kidney 293T cells. Consistently, miR-296-5p and miR-874-3p were demonstrated to decrease mRNA and protein levels of PIN1 in all 3 different cell lines (Figure 2B and 2C). To determine the function and specificity of miRNAs, specific miRNA inhibitors were co-transfected with their corresponding miRNAs in 293T cells. Expectedly, miR-296-5p and miR-874-3p inhibitors abolished the PIN1 down-regulation induced by their corresponding miRNAs (Figure 2D). These results showed that miR-296-5p and miR-874-3p specifically decreased PIN1 expression.

### The expression of miR-874-3p was significantly down-regulated in HCC and negatively correlated with PIN1

**Figure 3:**
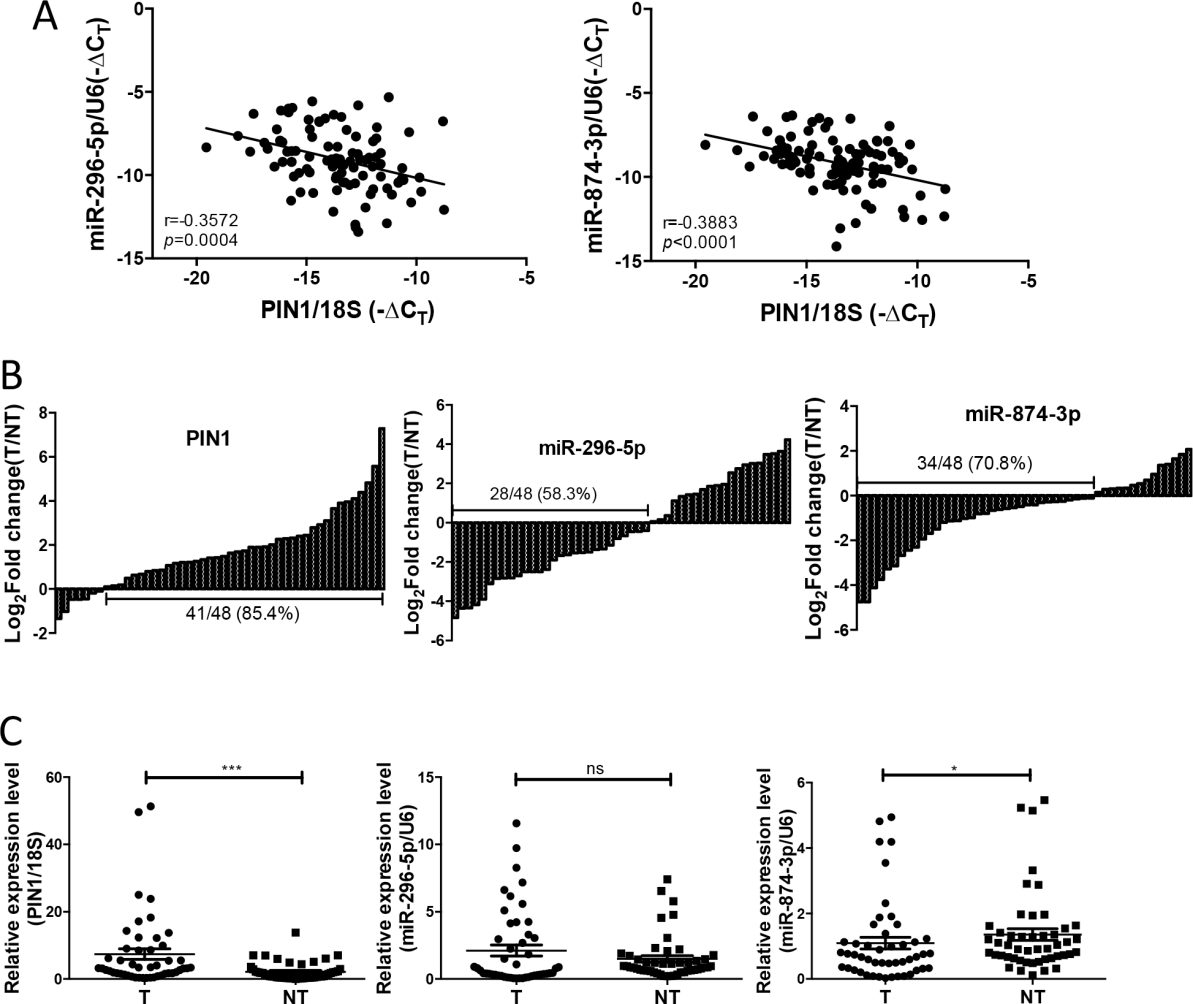
The expression of miR-874-3p was down-regulated in primary HCC tissues and correlated negatively with PIN1 (**A**) The expression of PIN1 was negatively correlated with miR-296-5p (left) and miR-874-3p (right) (**B**) The expression of PIN1 (left panel), miR-296-5p (middle panel) and miR-874-3p (right panel) for each paired sample was represented as log2 fold change of the tumour relative to the non-tumourous tissue (T/NT). (**C**) The relative expression of miR-296-5p, miR874-3p and PIN1 in tumour (T) and non-tumourous tissue (NT) were shown by scatter plot. (*P ≤ 0.05, and ***P ≤ 0.001; ns = no significance, Mann Whitney test).

**Figure 4:**
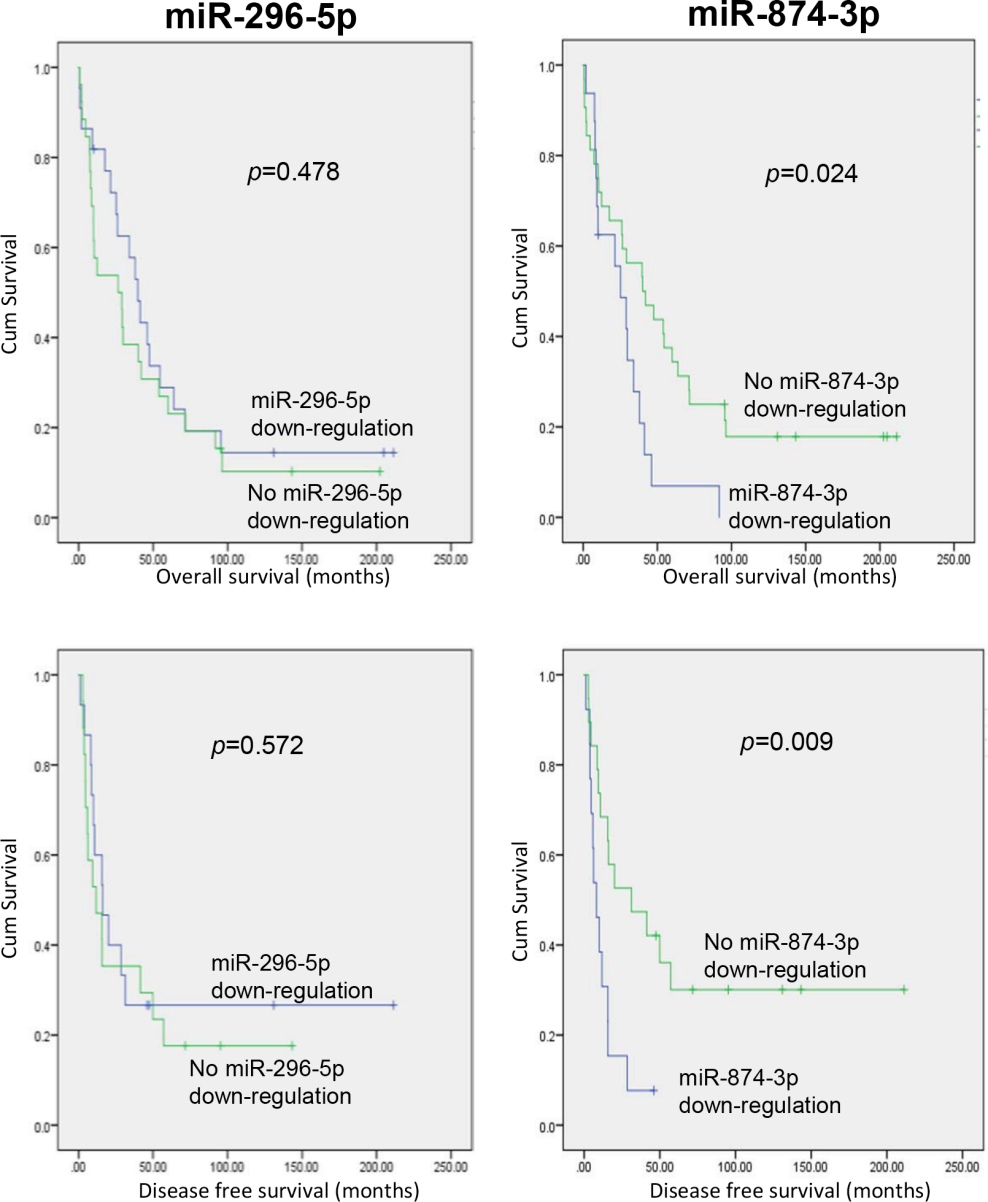
HCC patients with miR-874-3p down-regulation showed inferior overall and disease-free survivals Kaplan-Meier survival curves showing that HCC patients with down-regulation of miR-874-3p (right panels), but not miR-296-5p (left panels), exhibited lower overall (upper panels) and disease-free (lower panels) survival rates. P values were shown in the figure.

To evaluate the clinical relevance and significance of these regulatory miRNAs of PIN1, we analysed the expression levels of the 6 identified-miRNAs using the online HCC miR-Seq dataset of The Cancer Genome Atlas (TCGA) (http://cancergenome.nih.gov/). Only miR-296-5p and miR-874-3p were found to be detectable in HCC tissues and non-tumourous liver tissue (NT), whereas the other four miRNAs were not. Moreover, miR-874-3p, but not miR-296-5p, was significantly down-regulated in HCC while compared with NT (Supplementary Figure 2). We further examined the expression levels of miR-296-5p and miR-874-3p, and their correlation with that of PIN1 in 48 primary HCC samples and their paired NT. As shown in Figure 3A, miR-296-5p and miR-874-3p expression was negatively correlated with that of PIN1 (*p* = 0.0004 and *p* < 0.0001, respectively, Pearson analysis). The expressions of PIN1, miR-296-5p and miR-874-3p in HCC were further quantified in tumour samples and compared with their paired NT. PIN1 was found to be over-expressed in 85.4% of cases, whereas miR-296-5p and miR-874-3p were down-regulated in 58.3% and 70.8% of cases (Figure 3B). However, only miR-874-3p was demonstrated to be significantly lower in tumour, as compared with NT, consistent with the data obtained from TCGA database (Figure 3C). Correlation with clinicopathologic parameters showed that down-regulation of miR-874-3p was associated with poorly differentiated tumour cells and more advanced tumour staging (Table 1). More importantly, down-regulation of miR-874-3p, but not miR-296-5p, was also associated with inferior overall survival and disease free survival of HCC patients (Figure 4). Our results, therefore, suggested miR-874-3p to be a clinically and pathologically relevant negative regulator of PIN1, and might potentially play a tumour suppressive role in HCC.

**Table 1:** Clinicopathological correlation of down-regulation of miR-296-5p and miR-874-3p in HCC patients

			miR-296-5p downregulation			miR-874-3p downregulation		
		no. of patients	No	Yes	^*p*	No	Yes	^*p*
**Sex**	**Male**	38	18	20		27	11	0.4859
	**Female**	12	9	3	0.1119	7	5	
**Liver cirrhosis**	**Present**	28	11	17		22	6	
	**Absent**	22	16	6	**0.0244**	12	10	0.1256
**Tumour nodule**	**≥ 2**	10	6	4		7	3	
	**1**	40	21	19	0.7356	27	13	1
Cell differentiation*	**I–II**	19	5	14		17	2	
	**III–IV**	31	22	9	**0.0033**	17	14	**0.0134**
**Tumour size**	**> 5 cm**	37	21	16		23	14	
	**≤ 5 cm**	13	6	7	0.5369	11	2	0.1792
**pTNM stage^#^**	**I/II**	13	5	8		12	1	
	**III/IV**	34	20	14	0.3279	19	15	**0.0359**

*Edmondson grading.

^#^Pathological Tumor-Node-Metastasis stage.

^^^*p*-value calculated by Fisher's exact test.

### Direct binding of miR-874-3p to 3′UTR of *PIN1* mRNA

**Figure 5:**
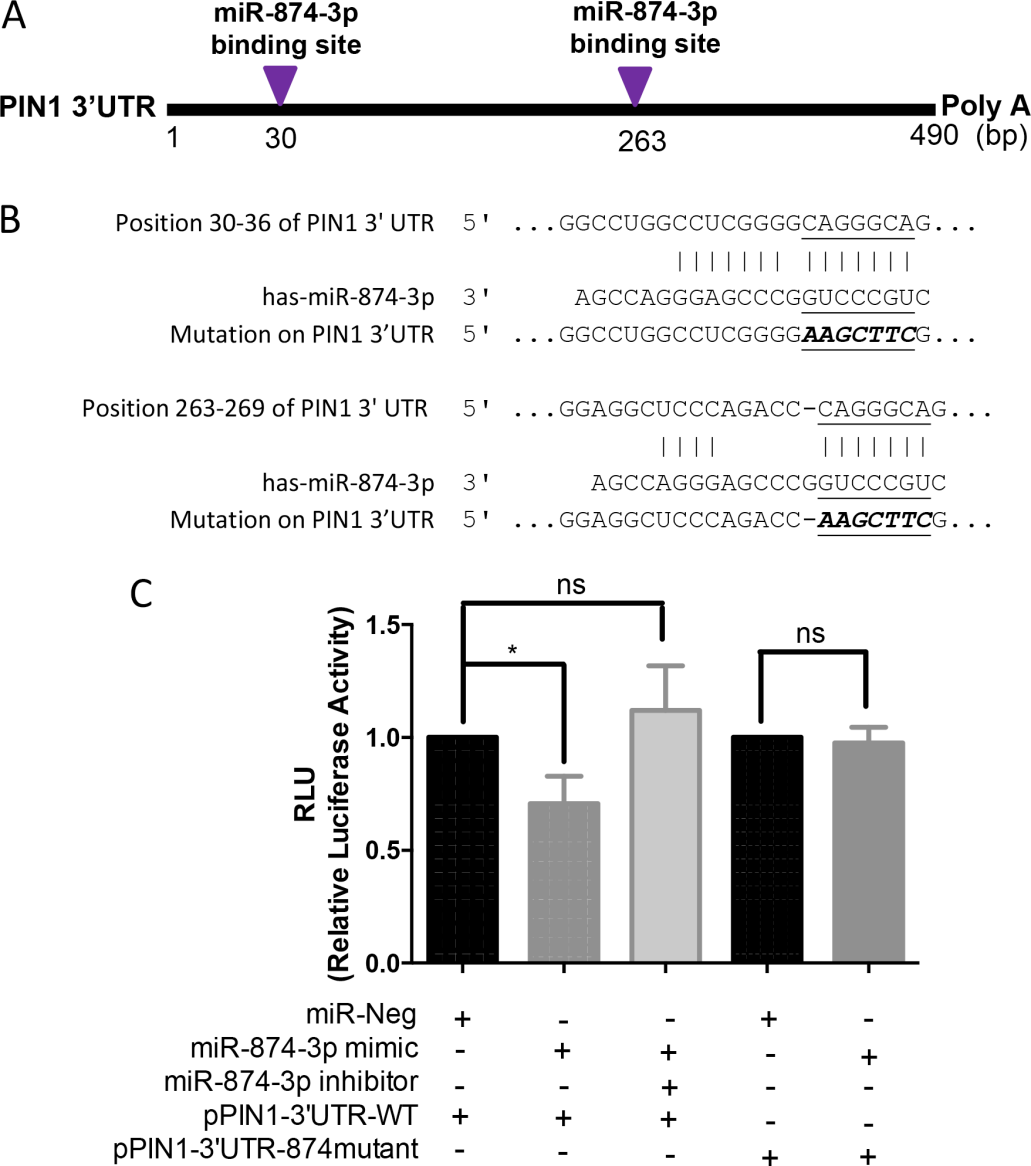
Direct binding of miR-874-3p to PIN1 3′UTR (**A**) Diagram showing the two predicted miR-874-3p binding sites on 3′UTR of PIN1 mRNA. (**B**) Sequences showing the putative miR-874-3p binding sites on PIN1 3′UTR and the mutated miR-874-3p binding sites generated by site-directed mutagenesis (**C**) Dual luciferase reporter assay showing significant suppression of luciferase activity only when miR-874-3p was partnered with wild-type PIN1 3′UTR, and miR-874-3p inhibitor restoring the decreased luciferase activity. The relative luciferase activities were normalized with Renilla activity and the miR-Neg control. Experiments were repeated in triplicates. (*P ≤ 0.05; ns = no significance, unpaired t-test).

To determine if miR-874-3p specifically bound to 3′UTR of *PIN1* mRNA, we examined the interaction between *PIN1* mRNA 3′UTR and miR-874-3p. There are two potential miR-874-3p binding sites in the *PIN1* mRNA 3′UTR (Figure 5A). Luciferase assays with plasmids containing *PIN1* mRNA 3′UTR that was wild-type (pPIN1-3′UTR-WT) and mutated at the miR-874-3p-binding-sites (pPIN1-3′UTR-874mutant) were performed (Figure 5B). As shown in Figure 5C, over-expression of miR-874-3p significantly reduced the luciferase reporter activity of pPIN1-3′UTR-WT but not pPIN1-3′UTR-874mutant. Moreover, concomitant expression of miR-874-3p specific inhibitor restored the inhibition of luciferase reporter activity of pPIN1-3′UTR-WT, showing that miR-874-3p bound to *PIN1* mRNA 3′UTR specifically to suppress PIN1 expression.

### Decrease in cell proliferation and *in-vitro* Experiments were repeated in triplicate scolony formation, and increase in apoptosis mediated by miR-874-3p in HCC

**Figure 6:**
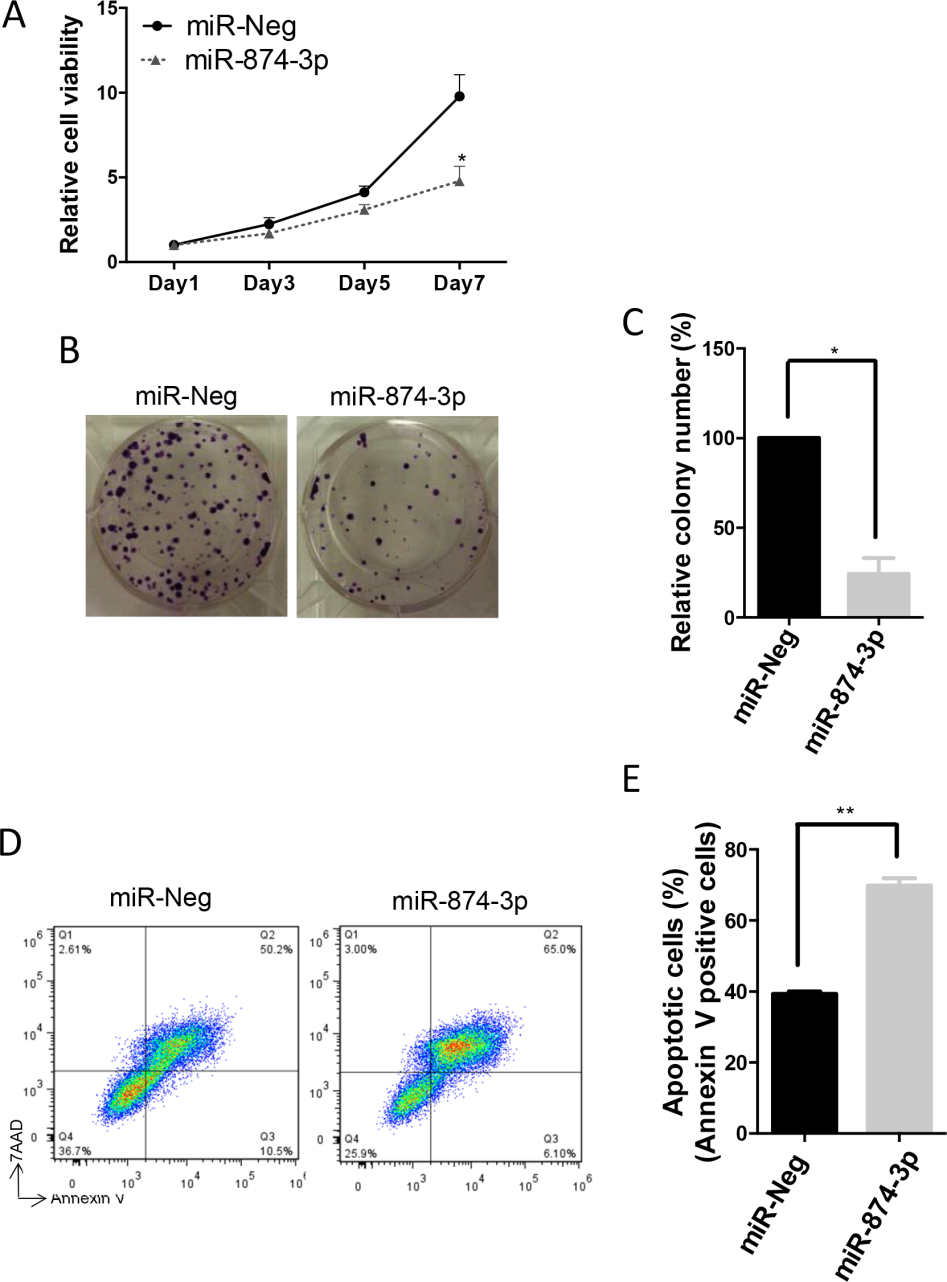
miR-874-3p suppressed cell proliferation and in-vitro colony formation, and enhanced apoptosis of HCC cells (**A**) Increased expression of miR-874-3p significantly suppressed cell proliferation of PLC/PRF/5 cells as determined by MTT assay. (*P ≤ 0.05, paired t-test) (**B**) miR-874-3p suppressed in-vitro colony formation of PLC/PRF/5 cells. (**C**) Diagram showing decreased in colonies formed by PLC/PRF/5 cells with miR-874-3p expression. (*P ≤ 0.05, unpaired t-test) (**D**) FACS analysis scatter plots showing increased apoptotic (annexin-V positive) cells with miR-874-3p expression. (**E**) Diagram showing increased numbers of apoptotic cells after STS treatment in PLC/PRF/5 cells with miR-874-3p expression. Experiments were repeated in triplicates. (**P ≤ 0.01, paired t-test).

To examine the functional significance of miR-874-3p in HCC cells, we performed MTT, *in-vitro* colony formation, and apoptosis assays in PLC/PRF/5 cells with or without miR-874-3p over-expression. Results from MTT and *in-vitro* colony formation assays showed that expression of miR-874-3p significantly inhibited proliferation and impaired colony formation of PLC/PRF/5 cells (Figure 6A–6C). Furthermore, miR-874-3p expression enhanced STS-induced apoptosis (Figure 6D and 6E). These results suggested that miR-874-3p assumed a tumour suppressive role in HCC cells.

### The tumour suppressive role of miR-874-3p was mediated by PIN1 down-regulation in HCC

**Figure 7:**
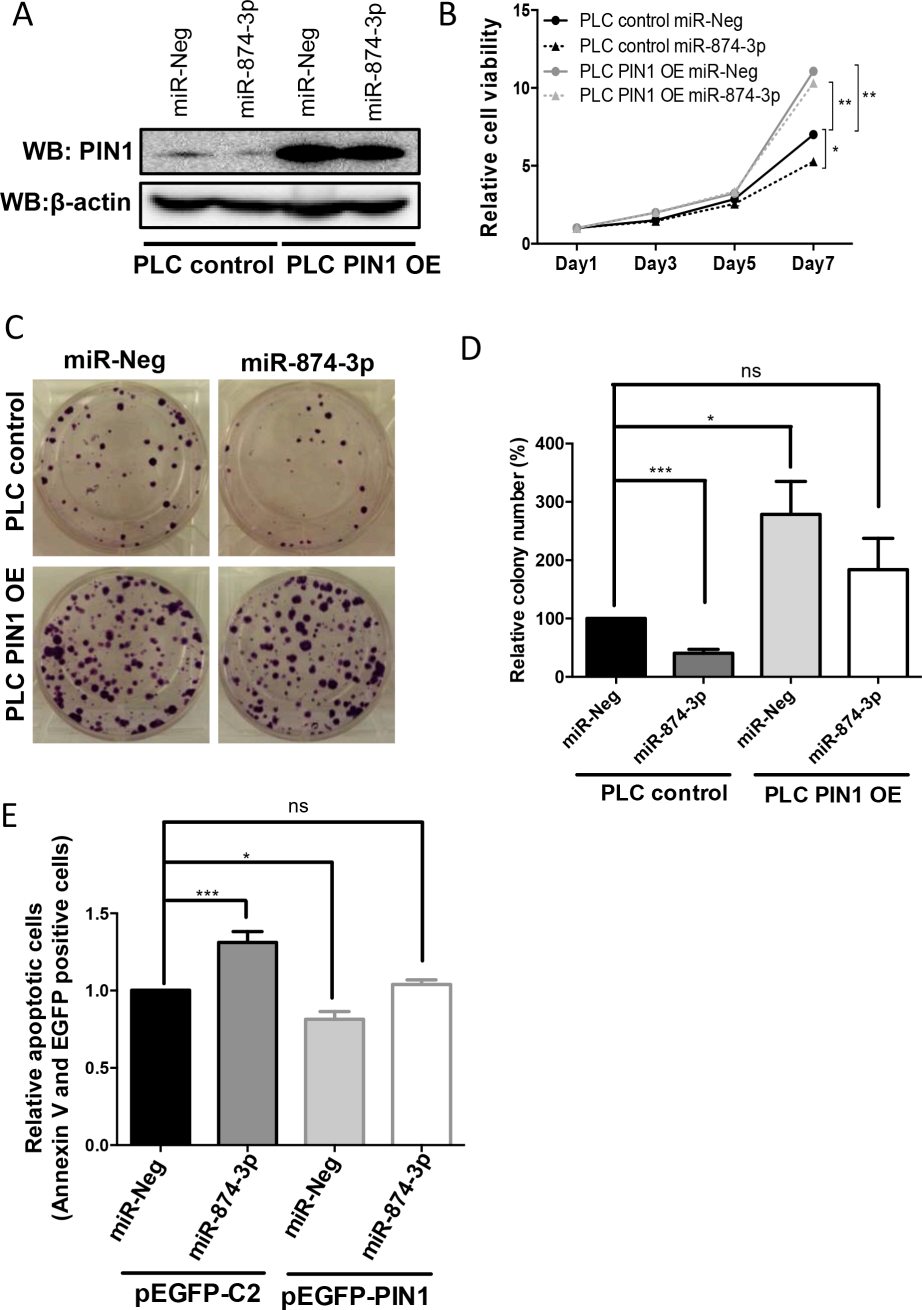
miR-874-3p regulated cell proliferation, colony formation as well as cellular apoptosis via down-regulation of PIN1 expression (**A**) Western blots showing the high level of PIN1 in PLC/PRF/5 cells stably transfected with PIN1 cDNA (PLC PIN1 OE) irrespective of miR-874-3p expression. (**B**) PIN1 over-expression abolished the suppressed cell proliferation mediated by miR-874-3p. (*P ≤ 0.05, **P ≤ 0.01, unpaired t-test) (**C**) PIN1 over-expression abrogated the suppressed colony formation mediated by miR-874-3p. (**D**) Diagram showing PIN1 over-expression ameliorated the inhibition of colony formation of PLC/PRF/5 cells by miR-874-3p. (**P ≤ 0.05, ***P ≤ 0.001, ns = no significance, unpaired t-test) (**E**) Diagram showing PIN1 over-expression suppressed the enhancement of cellular apoptosis mediated by miR-874-3p. (*P ≤ 0.05, ***P ≤ 0.001, ns = no significance, unpaired t-test).

To demonstrate that the tumour suppressive function of miR-874-3p was mediated through PIN1 down-regulation, PIN1 was over-expressed by *PIN1* cDNA transfection in PLC/PRF/5 cells with or without miR-874-3p expression. The level of over-expressed PIN1 protein in PLC/PRF/5 with *PIN1* cDNA transfection was not affected by miR-874-3p because the *PIN1* cDNA construct did not contain a 3′UTR, hence the transcribed *PIN1* mRNAs was not targeted by miR-874-3p (Figure 7A). In PLC/PRF/5 cells, PIN1 over-expression successfully abrogated miR-874-3p-mediated suppression of cell proliferation (Figure 7B). Similarly, the reduced *in-vitro* colony formation capability of PLC/PRF/5 with miR-874-3p expression was also reverted by PIN1 over-expression (Figure 7C and 7D). Furthermore, PIN1 over-expression also decreased cellular apoptosis that was enhanced by miR-874-3p expression (Figure 7E). These results showed that the tumour suppressive function of miR-874-3p was mediated through its down-regulation of PIN1. In addition, to further validate the role of PIN1 in miR-874-3p mediated tumour suppressive function, miR-874-3p was over-expressed in PIN1-silenced cells (PLC/PRF/5 with PIN1 knocked-down by siRNA). MTT assay showed that miR-874-3p over-expression did not suppress cell proliferation in PIN1-silenced cells, suggesting that PIN1 is an important downstream target of miR-874-3p (Supplementary Figure 3).

### miR-874-3p suppressed HCC cell growth *in vivo*

**Figure 8:**
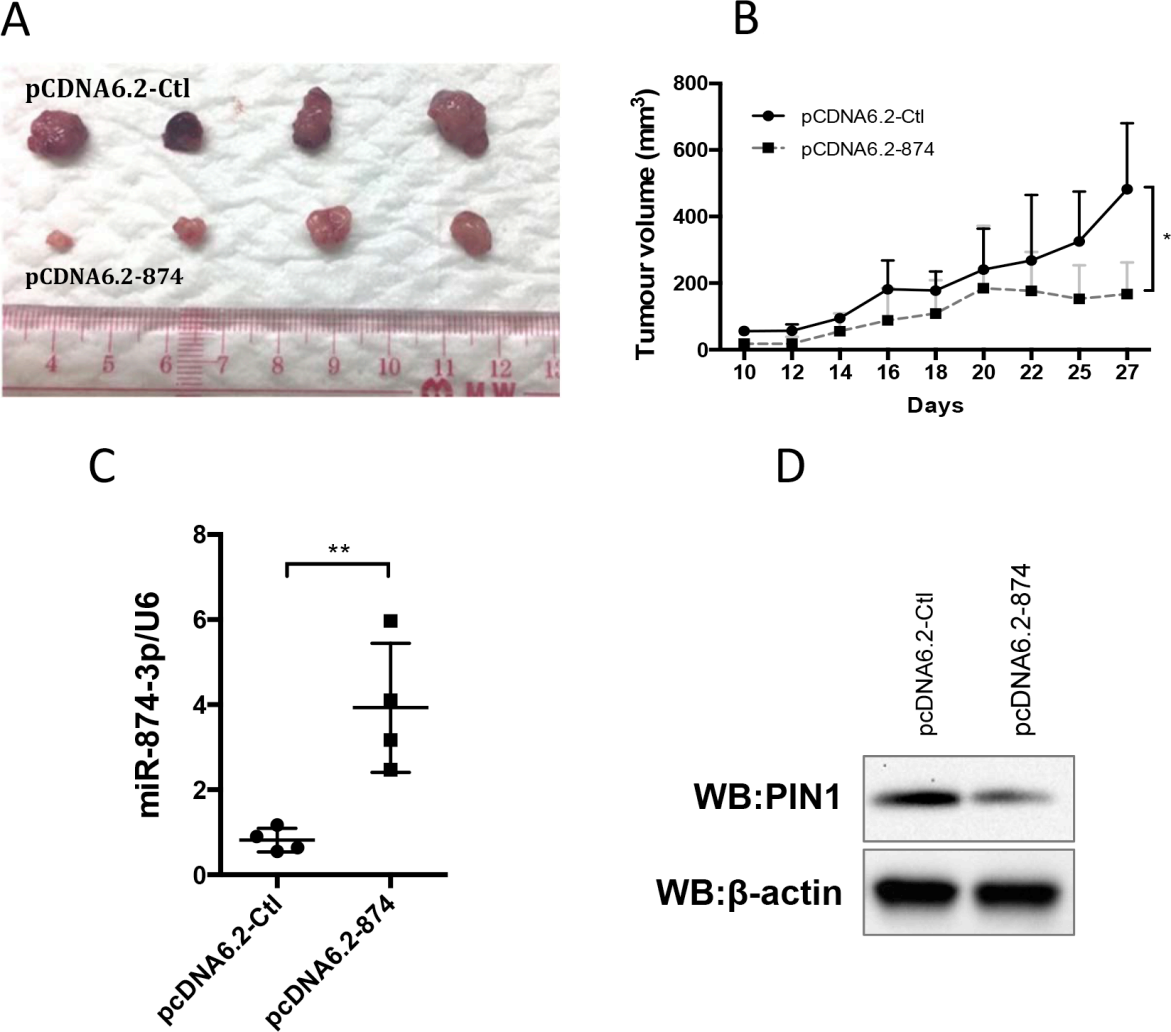
Expression of miR-874-3p suppressed HCC tumour growth in vivo (**A**) 2 × 106 PLC/PRF/5 cells with control sequence (pCDNA6.2-Ctl) or miR-874-3p (pCDNA6.2-874) over-expression were subcutaneously injected in the left flank and right flank of nude mice respectively. Tumour development was monitored for 28 days after injection. The size of tumours developed from miR-874-3p expressing cells was smaller than those of the control cells. (**B**) The development of tumours from miR-874-3p expressing cells was significantly slower. (**C**) RT-qPCR showing the higher miR-874-3p expression in the tumours derived from miR-874-3p expressing cells. (D.) Western blot showing the lower expression of PIN1 in one of the representative tumours derived from miR-874-3p expressing cells. (*P ≤ 0.05, **P ≤ 0.01, paired t-test).

We established a stable clone of PLC/PRF/5 cells expressing miR-874-3p to further examine the tumour suppressive function of miR-874-3p *in vivo*. Control sequence (pcDNA6.2-Ctl) or miR-874-3p (pcDNA 6.2-874) was cloned into pcDNA6.2 plasmid and stably expressed in PLC/PRF/5 cells. Cells were then subcutaneously injected in nude mice and the tumour growth was monitored. Consistent with the results of the *in vitro* experiments, PLC/PRF/5 cells with miR-874-3p expression formed significantly smaller tumours *in vivo*, as compared to the control (Figure 8).

## DISCUSSION

Despite the importance of miRNAs and PIN1 in the pathogenesis of HCC, the relationship between miRNAs and PIN1 in HCC has not been examined. In this study, we identified miR-874-3p as a tumour suppressive miRNA that down-regulated PIN1 expression in HCC. Although we found that miR-296-5p also decreased PIN1 expression, as was previously reported in prostate cancer, there was no differential expression of miR-296-5p between HCC and the corresponding non-tumourous liver tissues. Therefore, miR-296-5p might not directly contribute to the neoplastic phenotype. However, HCC typically evolves through a cirrhotic stage, meaning that the surrounding non-tumour tissues are also in fact abnormal. Hence, a role of miR-296-5p in the multistep process of hepatocarcinogenesis cannot be totally excluded.

In contrast, miR-874-3p was significantly down-regulated in HCC as compared with non-tumourous tissue. More importantly, down-regulation of miR-874-3p in HCC was associated with poorly differentiated tumour, higher tumour staging and inferior survivals, underscoring the tumour suppressive function of miR-874-3p. miR-874-3p has been consistently shown in various tumour types to be a tumour suppressive miRNA through regulation of STAT3, CDK9 and E2F3 expression [17–19]. On the other hand, miR-296-5p has been reported to function as both tumour suppressive and oncogenic miRNA through controlling the expression of p21 and PLK1 respectively [20, 21]. In HCC, our data suggested that miR-874-3p played a more critical role than miR-296-5p in carcinogenesis.

PIN1 is over-expressed in cancers of many organs, including breast, lung, and prostate, and is involved in carcinogenesis [22–24]. Our group has also shown that PIN1 is up-regulated in 50–70% of HCC [8, 10]. However, the control of PIN1 expression in HCC has not been previously investigated. Data in this report showed that miR-874-3p controlled PIN1 mRNA and protein expression through targeting of *PIN1* mRNA 3′UTR. Moreover, there was a significant negative correlation between PIN1 and miR-874-3p expression. Based on these observations, we propose that the high expression of PIN1 is partly contributed by the down-regulation of miR-874-3p during hepatocarcinogenesis. The mechanisms underlying the down-regulation of miR-874-3p remain to be defined.

Given the pathogenic role of PIN1 in HCC, PIN1 inhibition represents a novel approach for the treatment of HCC. PIN1 inhibitors such as juglone, PiB, and all-trans retinoic acid have been used successfully in *in-vitro* studies to inhibit tumour growth, supporting the role of PIN1 as a potential therapeutic target [25, 26]. In addition to small molecule inhibitors, tumour suppressive miRNAs are also potential anti-cancer agents [27]. The first miRNA mimic-MRX34 has already reached to the stage of phase 1 clinical study in patients with primary or metastatic HCC [28]. Observations in this study suggest that miR-874-3p may also be a potential therapeutic agent for HCC. This proposition warrants further laboratory and clinical evaluation.

## MATERIALS AND METHODS

### Cell culture and transfection

Human HCC cell lines, HepG2 and PLC/PRF/5, and human embryonic kidney 293T cells were cultured in Dulbecco's modified Eagle's medium (DMEM) (Life Technologies^TM^, MA, USA) with 10% fetal bovine serum (Life Technologies^TM^, MA, USA) and antibiotics (Life Technologies^TM^, MA, USA). All cell lines were grown in a humidified incubator at 37°C and supplemented with 5% CO_2_–enriched atmosphere. HepG2, PLC/PRF/5 and HEK 293T cells were seeded in antibiotic free culture medium for 24 hours, followed by transfection with 60 nM of siRNA (siNeg and siPIN1) (Qiagen, Germany), mimic microRNAs (miR-Neg, miR-296-5p and miR-874-3p) (Applied Biosystems^TM^, MA, USA) or microRNA inhibitors (miR-Neg, miR-296-5p and miR-874-3p) (Applied Biosystems^TM^, MA, USA) using Lipofectamine RNAiMAX (Life Technologies^TM^, MA, USA).

### HCC patient samples

Primary HCC tumour samples, each paired with its non-tumourous adjacent tissue, were obtained from surgical resection at Queen Mary Hospital, Hong Kong. Fresh tissues were immediately frozen in liquid nitrogen and stored at −80°C. The use of the clinical specimens for this study was approved by Institutional Review Board of the University of Hong Kong and the Hospital Authority of Hong Kong.

### Plasmids and site-directed mutagenesis

The construction of pEGFP-PIN1 and pCDNA-PIN1 has been described previously [10, 11]. Wild-type PIN1 3′UTR was cloned into the pmiR-reporter vector to generate pPIN1-3′UTR-WT. Primer sequences used for PCR of PIN1 3′UTR were as follows:

Forward: 5′- AGCTgagctcAGATGCAGAAGCCATTTGAAGAC-3′.

Reverse: 5′-AGCTaagcttCTTCCCTGAGGAGAAATGAGACA-3′.

Two sites on the PIN1 3′UTR were mutated in the pPIN1-3′UTR-WT to generate pPIN1-3′UTR-874mutant by site-directed mutagenesis (QuikChange II, Agilent Technologies, CA, US). Specific primers were designed (QuikChange Primer Design, Agilent Technologies, CA, US), and DNA sequences of all constructs were verified by direct sequencing.

Control oligo and miR-874-3p were cloned into pcDNA6.2 plasmid by BLOCK-iT Pol II RNAi Expression Vector Kits (Life Technologies^TM^, MA, USA). The sequences are as follows:

Control sequence:

5′-TGCTGAAATCGCTGATTTGTGTAGTCGTTTTGGCCACTGACTGACGACTACACATCAGCGATTT-3′.

miR-874-3p sequence:

5′-TGCTGCTGCCCTGGCCCGAGGGACCGGTTTTGGCCACTGACTGACCGGTCCCGGGCCAGGGCAG-3′.

### Establishment of PIN1 and miR-874-3p over-expressing PLC/PRF/5 stable clones

PLC/PRF/5 cells were transfected with pCDNA3.1(-) and pCDNA-PIN1 for 48 hours. Stable clones over-expressing PIN1 were selected after 2 week with 5 mg/ml G418 (Sigma-Aldrich, MO, USA) in DMEM medium and 10% fetal bovine serum (Life Technologies^TM^, MA, USA). Selected clones were expanded in 24-well plate and the protein expression of PIN1 was examined by western blot. For miR-874-3p, pcDNA6.2-874 plasmid was transfected into PLC/PRF/5 cells for 48 hours. The transfected cells were then selected by 20 ug/ml Blasticidin (Life Technologies^TM^, MA, USA) in DMEM medium and 10% fetal bovine serum (Life Technologies^TM^, MA, USA) for 2 weeks. Selected clones were then expanded and the expressions of miR-874-3p and PIN1 were examined by qRT-qPCR and western blot respectively.

### Reverse transcription and quantitative polymerase chain reaction (qRT-qPCR)

Total RNA was extracted by Trizol reagent (Life Technologies^TM^, MA, USA). For PIN1 and 18S rRNA, 0.5 μg RNA was reverse transcribed to cDNA by SuperScript^®^ III Reverse Transcriptase (Life Technologies^TM^, MA, USA). The resulting cDNAs were quantified by quantitative PCR with Sybr Green master mix (Applied Biosystems^TM^, MA, USA) and specific primers for PIN1 and 18S rRNA detection. The specific primers used for PCR were:

PIN1 forward: 5′-TCGCACCTGCTGGTGAA-3′.

PIN1 reverse: 5′- ACTGTGAGGCCAGAGAC-3′.

18S rRNA forward: 5′-AAACGGCTACCACATCCAAG-3′.

18S rRNA reverse: 5′-CGCTCCCAAGATCCAACTAC-3′.

For miRNAs, 10 ng RNA was used for target-specific reverse transcription (TaqMan MicroNRA Reverse Transcription Kit, Applied Biosystems^TM^, MA, USA). Levels of miR-296-5p, miR-874-3p and U6 snRNA were examined by TaqMan^®^ MicroRNA Assay (Applied Biosystems^TM^, MA, USA).

### Protein extraction and western blotting

Total protein was extracted with RAPI buffer (50 mM Tris-HCl, 150 mM NaCl, 1 mM EDTA, 1% (v/v) NP-40, 1X protease inhibitor cocktail and 0.25% (w/v) sodium deoxycholate, pH 7.4). Protein was size-fractionated by SDS-PAGE and transferred to nitrocellulose membrane (Bio-Rad Laboratories, CA, USA). The membranes were blocked with 1× TBST buffer (20 mM Tris pH 7.5, 150 mM NaCl and 0.1% Tween 20) with 5% w/v non-fat dry milk, incubated with specific primary antibodies at 4°C overnight, washed with 1× TBST, and then incubated with HRP-conjugated anti-mouse or anti-rabbit IgG antibodies for 2 hours at room temperature. The expression level of various proteins was detected by enhanced chemiluminescence (EMD Millipore, MA, US) and ChemiDoc Imaging system (Bio-Rad Laboratories, CA, USA). Anti-PIN1 (Calbiochem, CA, USA) and anti-actin (Sigma-Aldrich. MO, US) antibodies were used for western blots.

### MTT assay and *in-vitro* colony formation assay

Twenty hours after transfection, cells were seeded in 96-well plate one day prior to MTT [3-(4,5-dimethylthiazol-2-yl)-2,5-diphenyl-2H-tetrazolium bromide] assay. Cells were incubated at 37°C for 4 hours with 1 mg/ml MTT (Sigma-Aldrich, MO, USA) in DMEM medium with 10% FBS before measurement with CLARIOstar microplate reader at indicated days. For *in-vitro* colony formation assay, 0.5% crystal violet (Sigma-Aldrich, MO, USA) with 20% methanol was used to fix and stain cells 15 days after transfection.

### Luciferase reporter assay

HEK 293T cells were transfected with 60 nM microRNA mimics or inhibitors (Applied Biosystems^TM^, MA, USA) for 24 hours (Lipofectamine RNAiMAX, Life Technologies^TM^, MA, USA), followed by transfection with 10 ng of wild type or mutated PIN1 3′UTR reporter and 1 ng of Renilla plasmid for 48 hours (ViaFect transfection reagent, Promega, WI, USA). The luciferase activities were detected by Dual-luciferase reporter assay (Promega, WI, USA). Briefly, cells were lysed with 100 μl of 1X Passive Lysis buffer at room temperature and 20 μl of cell lysate was used for the detection of luciferase activity.

### Apoptotic cell analysis

PLC/PRF/5 cells were seeded a day before staurosporine (STS; 0.5 mM) treatment. Cells were stained with annexin and 7AAD by Annexin V-Phycoerythrin (Annexin V-PE) and 7-AAD Apoptosis Detection Kit (BD Biosciences, NJ, USA). The apoptotic cells were then detected by FACS with Cytomics FC500 (Beckman Coulter, CA, USA).

### *In vivo* xenograft experiment

Animal study was approved by the Committee on the Use of Live Animals in Teaching and Research of the University of Hong Kong. Human tumour Xenograft model was established in BALB/cAnN-nu (*nude*) mice (Charles River Lab, USA). 2 × 10^6^ PLC/PRF/5 cells with pcDNA6.2-Ctl or pcDNA6.2-874 stable expression were subcutaneously injected in the left flank and right flank of *nude* mice respectively. Tumours volume were measured every 2–3 days and were calculated as (L × W^2^)/2. L represented the length while W represented the width of tumour. After 28 days, mice were sacrificed and tumours were excised.

### Statistical analyses

Statistical analyses were performed with PRISM 5 software (GraphPad). Categorical data were analysed with χ^2^ test, and numerical data by *t*-tests. Survival analysis was performed with Kaplan-Meier method.

## SUPPLEMENTARY MATERIALS FIGURES


