# Supplementary material for: miR-874-3p is down-regulated in hepatocellular carcinoma and negatively regulates PIN1 expression
Source: Oncotarget. 2017 Jan 5;8(7):11343–55. doi: 10.18632/oncotarget.14526 (PMC5355269; doi:10.18632/oncotarget.14526)
Supplement: Supplementary file 1 [file oncotarget-08-11343-s001.pdf]

miR-874-3p is down-regulated in hepatocellular carcinoma and negatively regulates PIN1 expression

Supplementary Materials

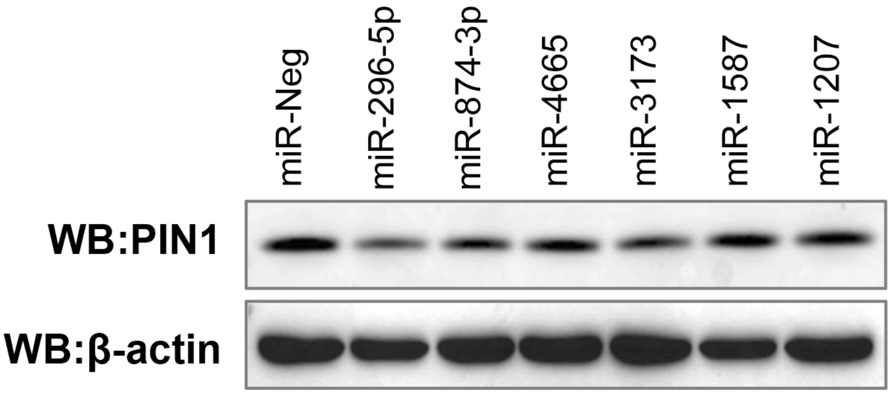

Supplementary Figure 1: Expression of miR-296-5p and miR-874-3p resulted in down-regulation of PIN1 expression in PLC/PRF/5 cells. The indicated miRNAs were expressed in PLC/PRF/5 cells and western blot was used to examine the PIN1 protein level. β-actin was used as an internal control. Only miR-296-5p and miR-874-3p significantly decreased PIN1 level in PLC/PRF/5 cells.

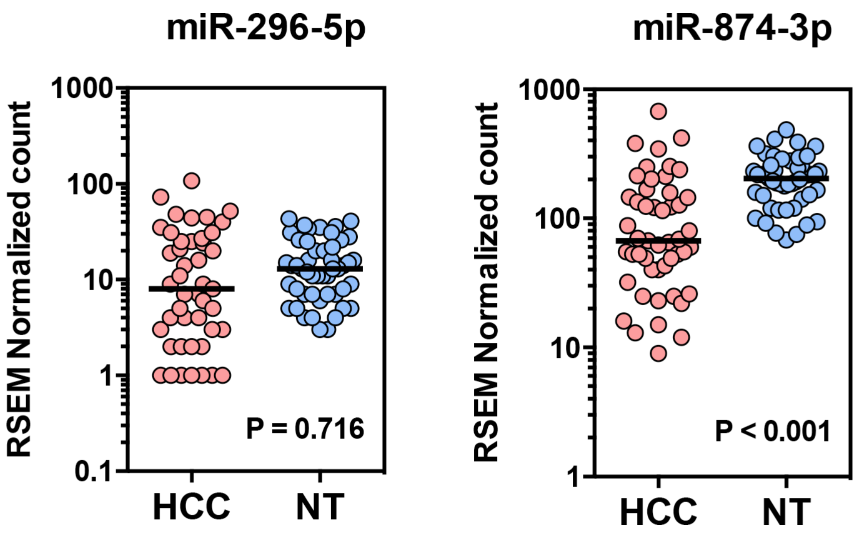

Supplementary Figure 2: miR-874-3p, but not miR-296-5p, was significantly down-regulated in HCC tissues as compared with non-tumourous tissue. The expression levels of miR-296-5p (Left panel) and miR-874-3p (right panel) were analysed in the 49 HCC cases from the TCGA database. Only miR-874-3p expression showed a significant decrease in HCC tissues (HCC) as compared with non-tumourous tissues (NT).

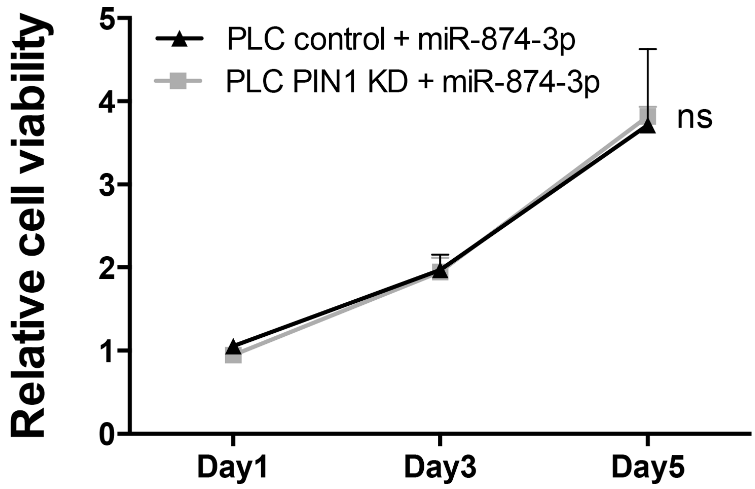

Supplementary Figure 3: MiR-874-3p expression did not suppress the growth of PLC/PRF/5 cells with PIN1 knocked-down. MTT assay was performed to examine the effect of PIN1 knock-down (PIN1 KD) in PLC/PRF/5 cells with miR-874-3p expression. MiR-874-3p expression did not suppress the growth of PLC/PRF/5 cells with silenced PIN1 expression.
